# Supplementary material for: An Optimized Small-Scale Rearing System to Support Embryonic Microinjection Protocols for Western Corn Rootworm, Diabrotica virgifera virgifera
Source: Insects. 2023 Aug 2;14(8):683. doi: 10.3390/insects14080683 (PMC10455090; doi:10.3390/insects14080683)
Supplement: Supplementary file 1 [file insects-14-00683-s001.zip › insects-2514613-supplementary.pdf]

Table S1. Reagents and Equipment

Name of Reagent/ Equipment	Company	Catalog Number	Comments/Description
30 ml (1 oz) Cup	Anny's plastic tableware	ASET101	Egg container
178 ml (6 oz) Plastic Souffle / Portion Cup	Webstaurantstore	128E506	Single pair mating chamber
475 ml (16 oz) Plastic Deli Container with Lid	Webstaurantstore	128hd16combo	Primary rearing container
950 ml (32 oz) Meal Prep Container	Webstaurantstore	128NC888	Secondary/Tertiary rearing container
Braided Cotton Rolls	Richmond Dental Cotton Co.	605-3599	Use in water supply for adult colony
BugDorm	MegaView Science	DP1000_5P	Cage for adult colony
Clorox Regular-Bleach1	Clorox	44600-30770	Washing corn and dishes
Cotton Balls, Large	Genesee Scientific	51-101	Capping the flask
Drosophila Agar	Apex	66-103	Making agar dish for egg-lay / type II
Falcon Tissue Culture Dishes	Falcon	25383-103	Sprouting corn / 150 x 25 mm
Featherweight Forceps	Bioquip	4748	Moving larvae and pupae
Fisherbrand Quantitative-Grade Filter Paper Circles	Fisherbrand	S47576C	Making agar dish for egg-lay / 9 cm
Globe Scientific 3.0 mL Small Bulb Transfer Pipettes	Globe Scientific	137035	Collecti and transfer eggs
Grade 90 Cheesecloth	Online Fabric Store	CHEE90	For egg-lay
Miniature Brush	Myartscape	MAS-102-MINI	Liner 2/0
Parafilm	Parafilm	734655769967	Seal agar dish after microinjection/roll size 4 in. x 125 ft
Percival Incubator	Percival	I41VLH3C8	WCR growth chamber
Petri Dish	SIGMA	CLS430588-500EA	Diet plate / 35 x 10 mm
Petri Dish	VWR	89038-968	Making agar dish for egg-lay / 100 x 15 mm
PYREX Griffin Low Form Beakers	PYREX	1000-600-PK	For washing eggs
PYREX Narrow Mouth Erlenmeyer Flasks	PYREX	4980-300-PK	Water container for adult colony
Qualitative Filter Paper	Ahlstrom	8613-0900	For microinjection / black
Reynolds Wrap Aluminum Foil	Reynolds	458742928317	Egg-lay cover
Scotts Premium Topsoil	The Scotts Company	71130758	Soil for growing corn
Sparkleen	Fisherbrand	04-320-4	For washing dishes
Trucker's Favorite Yellow	Coor Farm Supply	502	Corn for feeding WCR
Western Corn Rootworm w/o Pollen Substitute	Frontier Insect Diet	F9766B	WCR adult artificial diet

Table S2. Quality control raw data: survival rate

Week	# of Insects	# of Adults	Survival Rate %
1	264	204	77.27%
2	198	83	41.92%
3	589	447	75.89%
4	247	179	72.47%
5	293	240	81.91%
6	113	66	58.41%
7	346	261	75.43%
8	343	303	88.34%
9	519	459	88.44%
10	659	319	48.41%
11	489	354	72.39%
12	572	323	56.47%
13	263	215	81.75%
14	366	308	84.15%
15	375	129	34.40%
16	719	567	78.86%
17	491	456	92.87%
18	377	293	77.72%
19	432	278	64.35%

Table S3. Single-pair outcrosses to determine risk of pre-mating

Cross #	DsRed+ Larvae (week1)	Total Larvae	Total DsRed+	Total Larvae	Rate
1	4	29	53	412	12.86%
2	34	141	75	415	18.07%
3	43	146	121	432	28.01%
4	28	115	153	465	32.90%
5	54	118	66	149	44.30%
6	28	79	59	180	32.78%
7	23	42	30	68	44.12%
8	12	184	47	608	7.73%
9	22	200	24	239	10.04%
10	39	146	216	693	31.17%
11	8	97	37	463	7.99%
12	87	255	266	728	36.54%
13	28	111	107	636	16.82%
14	11	113	80	558	14.34%
15	57	167	163	469	34.75%
Control 1	61	139	85	211	40.28%
Control 2	58	133	87	268	32.46%
Control 3	38	105	70	272	25.74%
Control 4	22	104	41	244	16.80%

Outcrossed wild-type females with transgenic (DsRed) males to determine if pre-mating occurs in adult collection containers. Control crosses were transgenic females crossed to wild-type males.
